# Neurostimulation for cognitive rehabilitation in stroke (NeuroCog): study protocol for a randomized controlled trial

**DOI:** 10.1186/s13063-015-0945-1

**Published:** 2015-09-29

**Authors:** Suellen Marinho Andrade, Bernardino Fernández-Calvo, Paulo Sérgio Boggio, Eliane Araújo de Oliveira, Lilze Franklim Gomes, José Eudes Gomes Pinheiro Júnior, Rafaela Martins Rodrigues, Natália Leandro de Almeida, Gioconda Marla de Siqueira Moreira, Nelson Torro Alves

**Affiliations:** Cognitive Neuroscience and Behavior Program, Department of Psychology, Federal University of Paraíba, João Pessoa, Brazil; Cognitive Neuroscience Laboratory and Developmental Disorders Program, Mackenzie Presbyterian University, São Paulo, SP Brazil; Center for Research in Human Movement Sciences, Federal University of Paraíba, João Pessoa, PB Brazil; Perception, Neurosciences and Behavior Laboratory, Federal University of Paraíba, João Pessoa, PB Brazil; Department of Sciences of Religions, NOUS Group, Federal University of Paraíbas, João Pessoa, PB Brazil

**Keywords:** Transcranial direct current stimulation, Cognitive rehabilitation, Clinical trial

## Abstract

**Background:**

Stroke patients may present severe cognitive impairments, primarily related to executive functions. Transcranial direct current stimulation has shown promising results, with neuromodulatory and neuroplastic effects. This study is a double-blind, sham-controlled clinical trial aiming to compare the long-term effects of stimulation in two different cognitive regions after a stroke.

**Methods/Design:**

Sixty patients who suffer from chronic strokes will be randomized into one of four groups: dorsolateral prefrontal cortex, cingulo-opercular network, motor primary cortex and sham stimulation. Each group will receive transcranial direct current stimulation at an intensity of 2 mA for 20 minutes daily for 10 consecutive days. Patients will be assessed with a Dysexecutive Questionnaire, Semantic Fluency Test, categorical verbal fluency and Go-no go tests, Wechsler Adult Intelligence Scale, Rey Auditory-Verbal Learning Test, Letter Comparison and Pattern Comparison Tasks at baseline and after their tenth stimulation session. Those who achieve clinical improvement with neurostimulation will be invited to receive treatment for 12 months as part of a follow-up study.

**Discussion:**

Long-term stimulation could be analyzed in regard to possible adaptive changes on plasticity after structural brain damage and if these changes are different in terms of clinical improvement when applied to two important cognitive centers.

**Trials registration:**

Clinicaltrials.gov, NCT02315807. 9 December 2014.

## Background

Because cerebrovascular diseases are considered a major factor of mortality and morbidity in many parts of the world, new therapeutic methods have been proposed for the management of cognitive sequelae after a stroke. However, few studies have examined cognitive factors associated with treatment by neurostimulation after a stroke. More emphasis has been directed at functional improvement related to outcomes such as mobility, independence and daily living activities [1]. In contrast, it is estimated that 75 % of people will have losses in executive functions after a stroke. These difficulties cause problems in their abilities to perform day-to-day tasks and deal with other problems such as movement disorders, which are also derived from their brain injury [2].

Neurostimulation techniques such as transcranial magnetic stimulation (TMS) and transcranial direct current stimulation (tDCS) have shown promising results in this population [3]. Both techniques can lead to an improvement of lost skills/abilities, with virtually no side effects or discomfort for patients [4]. Regarding tDCS, there are numerous advantages compared to other non-invasive brain stimulation therapies, such as low cost, easy handling and high portability. During the application of electric current, essential neurostimulatory effects are observed. After stopping tDCS application, neuroplastic effects can be observed [5].

With regard to the stimulation site of cognitive areas, it is not yet clear what role each area would play during the planning and execution of cognitive information [6]. Although the dorsolateral prefrontal cortex (dlPFC) is considered the main cognitive control area used in experiments with tDCS [7], neuroimaging techniques have contributed to identifying other “multiple functional neural networks,” which may be related to processing or cognitive control categories [8]. A study involving mapping and interconnection of various regions showed that the frontoparietal network could serve as a “flexible hub” that would alter connectivity with other neural networks based on the specific task [9]. Other authors indicate that the parietal component seems to initiate and adjust the control, while the cingulo-opercular network (CON) provides a stable maintenance of the task, suggesting connections between two core networks and other weaker long-range connections between components [10].

In this respect, some aspects of cognition can be broadly generalized, and there is no agreed upon list of all components comprising executive functions (as an example). This results in discrepancies between the studies concerning which the factors should be related [11, 12].

To address the theoretical gaps, we propose that the cingulo-opercular network could serve as an alternative locus of stimulation for cognitive rehabilitation of these patients. Combined with this, this work represents the first controlled clinical trial involving the application of tDCS on stroke. It will include a follow-up of 12 months, making it possible to investigate not only the effects that occur in the short term, but also those that last for a longer period after neurostimulation. In a recent study that evaluated cognitive problems after strokes in young patients (<50 years), it was found that “mental slowness” occurs up to 10 years after brain injury, and so it is necessary to assess the presence of cognitive deficits after a long period of follow-up [13].

Thus, the objective of this study is to 1) evaluate the efficacy of tDCS on two different networks, the frontoparietal (specifically in the dlPFC) and the cingulo-opercular (anterior insula/frontal operculum), comparing them with each other for cognitive rehabilitation after a chronic stroke; 2) check whether there are differences between the benefits achieved by stimulation of these two cortical areas and how big these differences are; and 3) present the study protocol, the previous results of the clinical trial, ensuring adherence and compliance with the guidelines previously proposed.

## Methods/Design

### Design

A clinical double-blind, randomized, controlled trial will be performed involving patients with chronic stroke, randomly assigned to four groups: Group 1) active tDCS in fronto-parietal region (dlPFC); Group 2) active tDCS in the cingulo-opercular region-CON (anterior insula/frontal operculum); Group 3) active tDCS in the primary motor cortex (M1); and Group 4) sham tDCS in M1. This region was used as a place of active and placebo control.

**Fig. 1:**
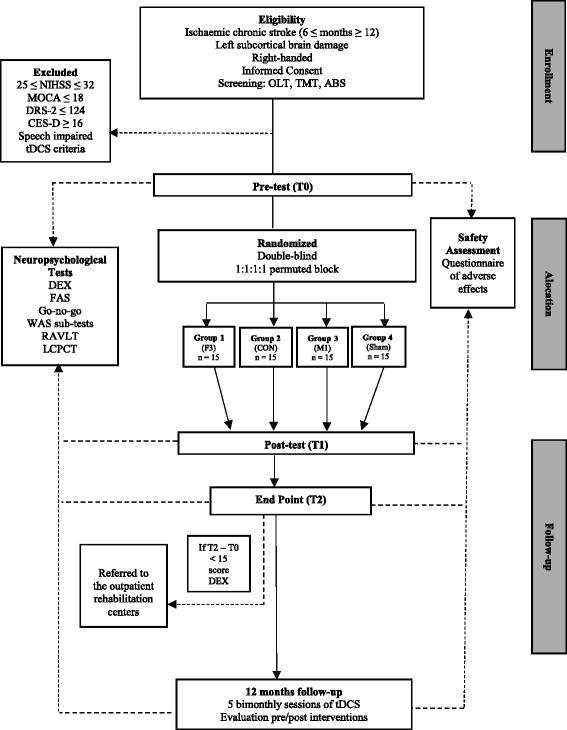
Flow chart of study. ASB: Assessment of Stroke and other Brain Damage; CES-D: Center for Epidemiological Studies-Depression; CON: cingulo-opercular network; DEX: Dysexecutive Questionnaire; dlPFC: dorsolateral prefrontal cortex; FAS: Semantic Fluency Test; DRS-2: Dementia Rating Scale-2; LCPCT: Letter Comparison and Pattern Comparison Tasks; M1: primary motor cortex; MOCA: Montreal Cognitive Assessment; NIHSS: National Institute of Health Stroke Scale; OLT: Object Learning Test; RAVLT: Rey Auditory-Verbal Learning Test; tDCS: transcranial direct current stimulation; TMT: Trail Making Test; WAIS: Wechsler Adult Intelligence Scale

Initially, all participants will undergo 10 consecutive daily sessions of neurostimulation (excluding weekends). Evaluations will be conducted pre- and post-test (T0 and T1, respectively). After 1 month, the participants will be checked to see who attained clinical improvement with treatment: that is, those patients who had a final score (T2) on the Dysexecutive Questionnaire (DEX) greater than at least 2.77 standard error of measurement compared from their baseline/initial testing [14]. Those who have an adequate response will be invited to receive treatment for 12 months. In this period of follow-up, the stimulation sessions will be applied twice monthly, for 5 consecutive days. This model is similar to another that has already been applied/used by other authors with positive results in a study involving tDCS for treatment of depression in order to assess whether the benefits of regularly performed neurostimulation continue in the long term [15]. Adverse effects will be computed periodically to prevent possible deleterious effects (see Fig. 1, which demonstrates the flow of the study). Previous studies have demonstrated that tDCS induces minimal discomfort sensations, such as mild tingling and itching sensations under the electrodes, predominantly in the first few seconds of the tDCS [16, 17].

The method of randomization will be 1: 1: 1: 1, with interchange between blocks generated by an online program (random.org). Concealed allocation will be used with numbered, opaque-sealed sequential envelopes so that the person responsible for allocation will not be in contact with patients or with the work of others. All examiners will be blind to the type of treatment the patient is receiving (site of stimulation) and other assessments that will be carried out. The efficiency of the masking/blinding mechanism will be evaluated at the time of the last interview with the evaluators. They will state what region they believe the patient received stimulation in. Patients will also be blinded with a masking evaluation carried out on the last post-test where they will try to guess in which region stimulation was applied: the main (dlPFC) or secondary area (CON) of cognitive control, the motor control region (M1) or sham stimulation.

Attrition will be considered when a) three alternating or two consecutive absences; b) presence of cognitive comorbidities throughout the trial; c) loss during follow-up. There will be adherence strategies administered such as being able to make up an absence the following week, and offering flexible schedules and frequent contact through phone calls confirming dates of procedures and reinforcing the importance of treatment compliance.

The study was approved by the Health Sciences Centre Ethical Committee (#30163714.0.0000.5188) and is governed according to the principles of the Declaration of Helsinki. To conduct the study, the informed consent of each participating patient will be collected. The protocol follows all the recommendations of the Standard Protocol Items: Recommendations for Interventional Trials Statement (SPIRIT) 2013 [18].

### Participants

Sixty right-handed patients will be selected, aged between 18 and 60 years, who have been diagnosed with stroke in the left subcortical ischemic of the middle cerebral artery territory; according to the International Classification of Diseases (ICD-10) and whose data have been obtained by computerized tomography or magnetic resonance. The occurrence of the injury within a 6 to 12 month period will be considered chronic [19]. Only those patients referred for cognitive rehabilitation based on the clinical diagnosis by the treatment team, including interviews and neuropsychological testing [20], will be included in the clinical study. The neuropsychological screen/testing will consist of the following tests [21]. The Object Learning Test [22] will be used to assess learning and short-time memory for visually presented material. The cutoff between normal and pathologic cognitive functioning is 36 points for patients ≤64 years. The Trail Making Test A [23], which will be used to assess psychomotor speed and attention, has a cutoff for normal and impaired cognitive function equivalent to 60 s. An Assessment of Stroke and other Brain Damage [24] will be used to evaluate motor apraxia, rational apraxia and visual-spatial ability. The present study will be set at 65 points for motor apraxia (lower quartile), 14 points for rational apraxia (full score), and 25 points for visual-spatial ability (lower quartile).

Exclusion criteria are as follows: inability to perform the tests due to losses in production or understanding speech; severe clinical status, assessed by the National Institute of Health Stroke Scale (NIHSS) ≥16 points [25]; severe cognitive comorbidities, diagnosed according to clinical evaluation and according to the Diagnostic and Statistical Manual of Mental Disorders, 4th. Edition (DSM-IV) [26], and supplemented by the following instruments: Montreal Cognitive Assessment (MOCA) ≤18 points [27], Dementia Rating Scale-2 (DRS-2) ≤ 124 points [28] , and the Center for Epidemiological Studies - Depression (CES-D) ≥ 16 points [29]. In addition, exclusion criteria for the use of cortical stimulation will be applied: a) use of drugs stimulating central nervous system activity; b) patients with implanted metallic or electronic devices; c) a cardiac pacemaker; d) patients who experience seizures/convulsions; and e) pregnancy.

### Intervention

A constant current stimulator (TCT Research Limited) will be used using electrodes of 5 × 5 cm^2^ embedded in saline (0.9 % NaCl) and application of 2 mA current for 20 minutes. The protocol is identical for placebo stimulation, but the current will stop after 30 seconds from the start of stimulation, a blinding method used in several previous studies [1, 15].

The positioning of the electrodes follows the 10–20 electroencephalogram (EEG) system model. The cathode will be positioned in the right supra-orbital region (contralateral to vascular injury), while the anode will have the following provision in the left hemisphere: group 1 (dlPFC), F3 position [30]; group 2, cingulo-opercular network (CON)-anterior insula/frontal operculum, the intersection point between T3-Fz and F7-Cz [31, 32]; and groups 3 and 4, M1 position [33].

All experiments will be conducted at the same time of day (afternoon shift), including stimulation and evaluation, to avoid possible circadian cycle influences.

### Procedures

The primary outcome of the study will be the Dysexecutive Questionnaire (DEX) [34], composed of 20 subtests, standardized, with total score ranging from 0 to 80 points, applied to the patient and a family member. The issues involve disturbances in executive functions such as emotional or personality, motivational, behavioral and cognitive changes. It is an instrument with ecological validity confirmed for patients with brain injuries and may provide important information on executive functions with implications for cognitive rehabilitation [35].

Secondary outcomes will be: the Semantic Fluency Test (FAS), which assesses the recall of words that begin with a certain letter and category fluency for the generation of words in a semantic class [36]; Go-no go, which tests self-monitoring, inhibition, initiation and cognitive flexibility [37]; Wechsler Intelligence Scale for Adults (WAIS), in the subtests of digit symbol coding, matrix reasoning, comprehension and letter-number sequencing for evaluation of executive functions [38]; Rey Auditory Verbal Learning (RAVLT) test that measures recent memory, learning, interference, retention and recognition memory [39]; Letter Comparison and Pattern Comparison Tasks [40].

### Safety

To control adverse effects, reports of patients with feelings/sensations of itching, tingling, burning, headache or other discomfort (1 none, 2 mild, 3 moderate, or 4 strong) will be recorded, along with whether this effect could be related to stimulation on a Likert scale; 1 (no relation) to 5 (strongly related).

If any damage/injury occurs to the participant, he or she will be offered medical assistance/treatment, physiotherapy and psychological care without any onus or cost to the participant.

### Statistical analysis

To determine the number of participants in each group, we will apply the proposed criteria for clinical trials. As observed in previous studies using the DEX for cognitive evaluation, we found that the average obtained by individuals with cognitive decline (without presence of dementia) undergoing rehabilitation protocols lies in a range of 15 to 17 points, with a deviation-pattern of 5.0 to 8.0 points [41–43]. The minimal importance difference will be considered based on the variation of the standard error of measurement approach, considering 1 for a small effect, 1.96 for a moderate and 2.77 for a large effect [14]. From these criteria, considering 90 % power and taking into account possible dropouts, we calculate a final sample size of 90 participants. A significance level of *P* <0.05 will be considered.

An analysis of intention to treat will be used, and sensitivity analysis will be performed with different allocation procedures to verify the strength of the data. The best strategy resulting from the comparison of the following methods will be chosen: last observation carried forward, complete-case analysis, likelihood-based methods and multiple imputation.

For the primary endpoint, we will use a mixed linear model. We will model the change in cognitive performance according to the DEX using the covariates of time, group and interaction between treatment and time. The covariance with repeated measures and for each patient will also be measured. For secondary outcomes, repeated measures analysis of variance (ANOVA) will be used in which the dependent variable is the performance in each cognitive test and the independent variables are group (dlPFC, CON, M1, or sham), treatment time (baseline/initiation, week 2 and follow-up) and interaction group × treatment time. If necessary, post-hoc comparisons using Bonferroni correction will be made.

Deleterious and adverse cognitive effects will be calculated in terms of the proportion in each group and in each period, and will be analyzed by the Fisher exact test.

## Discussion

The primary objective of the study is to determine whether stimulation of the dlPFC and CON affects cognition in patients after a stroke. In addition, we seek to examine whether these effects are similar to each other and what is the clinical significance of the identified changes. Although studies with noninvasive stimulation focused on patients with cognitive dysfunction are important, completing a long-term follow up of participants who suffer strokes is a possibility for new advances in terms of prognosis. Traditionally, they would only have access to motor rehabilitation.

Some work has been published in order to explore the role that these two dlPFC and CON networks play [44]. However, clinical evidence related to the function of these two areas in terms of strategies for decision-making and other real life situations has been compromised [45]. In order to compare the effects of neurostimulation at these two sites, we propose to identify recovery markers through standardized assessment protocols.

In general, the strengths of our study include the following: 1) proposing a new approach of different stimulation from the classical cognitive approach after a stroke; 2) conduct longitudinal monitoring of responses to therapy, measuring potential neuroplastic benefits; 3) employing rehabilitation that is easy to use, low cost, safe and feasible; and 4) characterize cognitive markers as predictors of response.

Nevertheless, we can cite the insertion of chronic patients as a constraint, which limits the generalization to other phases of the stroke. Because there are cognitive and sensorimotor differences in the various phases of a stroke, it was decided to choose one of them, for greater control of the data. This, however, does not preclude the conduct of future studies that compare patients in different stages in order to know at what time after the injury stimulation could be more effective. Furthermore, the treatment will be performed in the laboratory, which can result in losses or withdrawals by participants. To minimize these effects, adherence strategies will be employed (direct and frequent contact with patients, scheduling flexibility and reinforcement of the importance of care continuity).

Another important aspect is that during the repeated measures application of neuropsychological tests, ceiling effects may occur, which will reduce the longer the interval between assessments; conversely, there will be a greater risk of changes in the characteristic being measured. In this study, the constructs analyzed did not express significant volatility over time, and the assessment range was based on standardization applied in previous studies involving neurostimulation and cognitive assessment [33, 46]. Thus, our study provides important implications for rehabilitation, both in the theoretical aspect to support new trials and also in a clinical setting to provide evidence to be applied in treating patients.

## Trial status

Recruitment and interventions are in progress.

## Abbreviations

ANOVA: analysis of variance
CES-D: Center for Epidemiological Studies - Depression
CON: cingulo-opercular network
DRS-2: Dementia Rating Scale-2
DSM-IV: Diagnostic and Statistical Manual of Mental Disorders
dlPFC: dorsolateral prefrontal cortex
DEX: Dysexecutive Questionnaire
EEG: electroencephalogram
ICD-10: International Classification of Diseases
MOCA: Montreal Cognitive Assessment
NIHSS: National Institute of Health Stroke Scale
M1: primary motor cortex
RAVLT: Rey Auditory Verbal Learning
FAS: semantic fluency test
SPIRIT: Standard Protocol Items: Recommendations for Interventional Trials
tDCS: transcranial direct current stimulation
TMS: transcranial magnetic stimulation
WAIS: Wechsler Intelligence Scale for Adults
